# Did organs precede organisms in the origin of life?

**DOI:** 10.1093/femsml/uqae025

**Published:** 2024-12-23

**Authors:** Fernando Baquero, Gabriel S Bever, Victor de Lorenzo, Val Fernández-Lanza, Carlos Briones

**Affiliations:** Division of Biology and Evolution of Microorganisms, Ramón y Cajal Institute for Health Research (IRYCIS), 28034 Madrid, Spain; Network Medical Research Center for Epidemiology and Public Health (CIBERESP), 28029 Madrid, Spain; Center for Functional Anatomy & Evolution, Johns Hopkins University School of Medicine, Baltimore, MD 21205, United States; Systems Biology Department, Centro Nacional de Biotecnologia, CSIC, 28049 Madrid-Cantoblanco, Spain; Division of Biology and Evolution of Microorganisms, Ramón y Cajal Institute for Health Research (IRYCIS), 28034 Madrid, Spain; Network Medical Research Center for Infectious Diseases (CIBERINFECT), 28029 Madrid, Spain; Bioinformatics and Biostatistical Research Unit, Ramón y Cajal Institute for Health Research (IRYCIS), 28034 Madrid, Spain; Department of Molecular Evolution, Centro de Astrobiología (CAB), CSIC-INTA, Torrejón de Ardoz,28864 Madrid, Spain

**Keywords:** proto-organs, organs, organisms, origin of life, reproduction, molecular evolution

## Abstract

Evolutionary processes acting on populations of organized molecules preceded the origin of living organisms. These prebiotic entities were independently and repeatedly produced [i.e. (re)-produced] by the assembly of their components, following an iterative process giving rise to nearly but not fully identical replicas, allowing for a prebiotic form of Darwinian evolution. Natural selection favored the more persistent assemblies, some possibly modifying their own internal structure, or even their environment, thereby acquiring function. We refer to these assemblies as proto-organs. In association with other assemblies (e.g. in a coacervate or encapsulated within a vesicle), such proto-organs could evolve and acquire a role within the collective when their coexistence favored the selection of the ensemble. Along millions of years, an extraordinarily small number of successful combinations of those proto-organs co-occurring in spatially individualizing compartments might have co-evolved forming a proto-metabolic and proto-genetic informative network, eventually leading to the selfreplication of a very few. Thus, interactions between encapsulated proto-organs would have had a much higher probability of evolving into proto-organisms than interactions among simpler molecules. Multimolecular forms evolve functions; thus, functional organs would have preceded organisms.

## Introduction: reproduction and (re)production

This hypothesis paper focuses on an alternative view of the development of processes that resulted in the emergence and early evolution of life by natural selection. The canonical identification of natural selection as concurrent with life is useful to operatively define a “living being,” which is conceived as an individual entity able to acquire and convert energy (metabolism), store information (genotype), and convert it to a phenotype, and as a result transfer this information to a new generation (reproduction). For Darwinian evolution to occur, reproduction must include the possibility of variation among the offspring and further selection of the fittest individual. What we proposed here is that the components of this process originated and evolved prebiotically. Indeed, this implies a different view of the concept of iterative selfreproduction as the main hallmark of life (Trifonov 2011). In the classic view, reproduction is the process by which organisms replicate themselves, occasionally giving rise to a more or less heterogeneous progeny that can be subjected to Darwinian evolution. Progressing in a top-down analytical direction, here we face the epistemological problem of reproduction versus iterative production or what we call (re)production. Assemblies are (re)produced spontaneously condensation from their constituents, rather than reproducing themselves (Bapteste and Papale 2021). During the (re)production, new elementary pieces can be captured and eventually added or replaced with other similar pieces in the molecular assembly, thus offering new variants to be tested by natural selection, in this case, not requiring conventional reproduction (Bourrat 2014). Some of them will be selected because of their resistance to their inevitable entropic breakdown. Individual differences in this stability would constitute the “variation” requirement of an evolutionary system, so that complex networks/ensembles can, in fact, be considered units of selection (Doolittle and Inkpen 2018).

The independent interactive networks submitted to evolutionary processes are called “evosystems” (Papale et al. 2024): a (bio)molecule or an ensemble of them that ensures that a long period of existence will be naturally selected over more labile or ephemeral structures. In addition, the parts composing an ensemble or network might be more protected against degradation than if they were elementary, independent entities. This concept of “resistance to entropy” in complex prebiotic systems is based on the building-up of an ordered system, thus limiting (or reducing in time) the number of possible ephemeral microstates and allowing the selection of those with higher durability, persistence over time (Dussault and Bouchard 2017). In this view, the ultimate role of natural selection (even in the prebiotic context) is to ensure existence over time (persistence), given that the more stable molecular individuals, will enrich the environment with their components, thus facilitating its (re)production from these components. This might represent a trend where (re)production could have later evolved to reproduction.

## Prebiotic natural selection

What precedes the natural selection of living organisms? Why are some molecular assemblies selected against others? The existence of discrete, relatively stable entities is necessarily anti-entropic. In other words, the existence of such assemblies is due to their selfpreservation. The term (re)production only means that an entity is *produced* several times. Atom–atom interactions independently *produce*, by Goethean “elective affinities,” particular inorganic (such as salts, carbon dioxide, or water, among many others) or organic (including bio monomers or other low MW compounds) molecules, with the same (thermodynamically and kinetically favored) reaction able to be independently (re)produced in various physicochemical environments. Iterative (either spontaneous or chemically catalyzed) (re)production, such as molecular assembly or template-free polymerization of monomers, creates “order” and favors the interaction of these elementary components in more complex and stable assemblies, in particular, environmental sites. In turn, the natural processes that give rise to “physical compartments” (phase differentiation in insoluble colloids and coacervates, or micelles and vesicles with different shapes, among other structures) also offer a way of assuring the local persistence of composing molecules (Hanczyc et al. 2003). Indeed, the conceptual core of natural selection is permanence over time: we can say that time itself is sequestered by order, the existential anti-entropic force, or the existence-associated energy (Baquero 2005, Johnson 2014).

As noted earlier, natural selection implies the existence of individuals (giving rise to units of selection) embedded in populations of other (more or less similar) individuals. In prebiotic natural selection, driven by stability and (re)production, we can imagine those individuals as biomolecules (e.g. RNA oligomers or peptides) and ensembles of them (e.g. coacervates or filled vesicles). (Cooper and Klymkowsky 2005, Ruiz-Mirazo et al. 2014, Agrawal et al. 2024). At these early stages of evolution, individuals evolved at distinct levels of selection; thus, stable “assemblies of molecules” could be considered individuals of higher hierarchical order with respect to the molecules themselves. This view of the possibility of selection on natural assemblies of prebiotic molecules in proto-living systems was strikingly described by Malaterre (2010) as “less-than-living” yet “more-than-non-living” ensembles. What we propose is that some of these assemblies might be converted into primordial functions, paving the path for the future emergence of organs and organisms.

## The dawn of functions

The hypothesis proposed in this work is that molecular assemblies, endowed with particular structures and compositions, acquire functions (including selfpreservation) which when collected and combined produce proto-organs, organs, and organisms. The novelty of such a perspective is the consideration that the spatial combination of early organs, and not lower chemical entities, should, by probabilistic reasons, precede (determine) the emergence of organisms. Similarly, in a backward-looking approach, functions “found” by assemblies of molecules precede (determine) proto-organs and organs. The notion of function is polysemic (Wouters 2003). However, order necessarily produces forms or shapes, and forms are a precondition for functions The classic “FFF” motto, “form follows function” illustrates such a point (Russell 1982). For some structural features, certain functions are difficult to separate from the forms that produce them: spheres protect and ensure the maximum surface-to-volume ratio, hexagons completely cover flat surfaces, spirals ensure packing, helixes bind, and chains endure (Wagensberg 2008). We propose that the notion of “functional anatomy” (in a sense, the classical FFF) should not be limited to research in animal or plant evolution (Johansson et al. 2005), but is also critical for the explanation of the evolution of structures and functions in other biotic and even prebiotic worlds. This “functional anatomy” also has a prebiotic role, giving rise to a “preparatory metabolism” (Fry 2011).

At the early stages of evolutionary processes, nonfunctional assemblies will vastly outnumber functional ones. However, given the wealth of possible molecular combinations in a virtually unlimited space of interactions, a particular molecular assembly or aggregate might approve functional. As in all fields of biology, functions were not teleonomically projected, but emerged by the molecular assemblies due to a successful combination of Monodian chance and necessity (Monod 1971). Complexity, and eventually functions, can arise through a series of nonadaptive steps, as proposed by the hypothesis of “constructive neutral evolution” (Muñoz-Gómez et al. 2021). Accordingly, a function can be considered aas ny activity, not necessarily resulting from natural selection, associated with a form that is able to change the environment of an individual in response to a physicochemical alteration. Eventually, such a change can result in benefits for the molecular entity, such as greater opportunities to be formed, greater stability over time, or greater reactivity. From this point, the emerging functions resulting from natural selection can provide more efficiently selectable effects for the individual (Amundson and Lauder 1994). At this prebiotic stage, form and function evolved together, following trade-offs between function and form optimization, pushing integrated functions into more consistent (robust) ensembles of growing hierarchies, which were finally consolidated by the birth of particular “functional forms.”

Individual functional forms without any organ linkage do not produce “ecology,” which implies biodiversity and ecosystem function. They are, in this sense, “isolated,” working only for their own existence and persistence in time. Reinterpreting Dussault and Bouchard' (2017) words, there can be a dissociation of the concept of function from evolutionary considerations, entailing “ahistorical functions”; this is, indeed, a “backward-looking approach to functions.” These authors also emphasize the notion of “persistence” (persistence enhancing propensity) as a fundamental ingredient in evolution, eschewing reproduction, and lineage formation as conditions for natural selection (Dussault and Bouchard 2017). This concept, even if rooted in structured forms as living things (Bouchard 2011, 2014), is close to the one used in this work (“existential force”), assuring the permanence of any entity in time, which does not exclude “differential persistence,” playing a similar role to “differential reproductive success” in evolutionary biology. In fact, our proposal approximates the concept of “function” as conceived from evolutionary thinking and froma purely ecological perspective, that is, describing what a system does in the context of surroundings, in our case, the early molecular environment.

The notion of proto-organs involves investigating the difference, at the level of molecular assemblies, between individualism (the structure works for itself) and utilitarianism (the structure works for the good of a complex whole) (Wagoner 2021). Although the function found by proto-organs only favors their individual existence, when selected in a given environment it can evolve to work in more complex, cooperative, more persistent wholes, successively following multilevel selection phases 1 and 2 (MLS1 and MLS2, Damuth and Heisler 1988). At this transition, proto-organs would become organs.

## Functions and organs

Etymologically, the word “organ” comes from the Ancient Greek “oργανον,” whose original meaning was “something that serves as a tool or instrument.” Therefore, an organ is simply something that performs a function. In our context, “organismic biology” refers to the parts with different but vital functions that make possible the existence of a biological individual, the “organism,” which is composed of the totality of interacting organs. Although the precise definition of organism(s) is still a matter of discussion, it remains a central concept in biology (Pepper and Herron 2008). Its seminal conceptualization was proposed in 1919 by the zoologist William E. Ritter, following the track of the evolutionary biologist John S.B. Haldane: “The organism in its totality is as essential to an explanation of its elements as its elements are to an explanation of the organism” (Ritter 1919, Herring and Radick 2019). Any organism requires established, effective, and regulated trade-offs among its organs, that is, an *organ*ization. In the best-known example, multicellular organisms have (more or less complex) organs (Minelli 2021). In turn, all cells have “organelles,” the equivalent of organs at the sub-cellular level. This concept was probably intuited by Anton van Leeuwenhoek in his first description of microbes as little animals (“*kleine dierkens*”) or “animalcules” on translation, when he observed them under the microscope (van Leeuwenhoek 1677), thus assuming the presence of organs within them (Gest 2004). Later on (∼1880), “microorganism” was Louis Pasteur's preferred denomination for unicellular organisms, based on the term “microscopic organisms” used by the French surgeon Charles Sédillot (Cavaillon and Legout 2022). In 1884, the German zoologist Karl August Möbius used the terms “organulum” (the diminutive of the Latin “*organum*”) and the plural “organula” for the first time, to refer to the various parts of the cell (Möbius 1884). In modern times, “organula” is translated as “organelles.” However, regardless of their size or complexity, organelles and organs are conceptually and ontologically equivalent. Therefore, throughout this work, we will use the term “organ” for both of them, while “organism” will be used for both multicellular and unicellular ones.

## The evolution of proto-organs precedes the evolution of organs

The origin of proto-organs (as defined above) and the functional interactions among them is currently explored by prebiotic systems chemistry within the field of the origin(s) of life (Ruiz-Mirazo et al. 2014, de la Escosura et al. 2015, Fox et al. 2015). What we would like to emphasize here is that such primitive functional associations among molecules and molecular assemblies initially formed simple organ-like structures (proto-organs), before their integration into proto-cells. Indeed, a “life-like system of organs but without cells” could have existed, thus progressing in the “evasion from the decay to equilibrium” postulated for living matter by the physicist Erwin Schrödinger (Schrödinger 1944, Pascal et al. 2013). This view is compatible with the more recent biophysical hypothesis proposed by Jeremy L. England, who suggests that random groups of molecules can selforganize to more reliably absorb and dissipate heat from the environment and that such selforganized systems preceded selfreplicating entities and are an inherent part of the physicochemical world (England 2013). However, such a hypothesis did not include the concept of assemblies of proto-organs that we are emphasizing as the origin of cellular life.

We can thus imagine a “communal non-cellular prebiotic scenario” in which all available functions are present and interact extensively among them, probably in a stochastic manner, as was presumed by Woese (2002). Some of these associated functions could have led to the independent (re)production of the communal, proto-biological system. Then, the most efficient (re)productive prebiological assemblies (proto-organs) in the communal system were selected, including those tightly associated with protective inorganic (mineral or metal) surfaces (Pérez-Aguilar and Cuéllar-Cruz 2022) and progressively evolved toward a true reproduction. At a critical moment (perhaps the first major transition in evolution, following the concept coined by the theoretical biologist John Maynard Smith (Maynard-Smith and Szathmáry 1995), a particularly lucky association of proto-organs acquired the capability of selfreproduction, such as Woese's “progenote.” Moving from (re)production to reproduction required that a genetic molecule and a metabolism be combined in the same compartmentalized system. This combination guaranteed the evolutionary continuity of a proto-cell and therefore of the repertoire of organs that proved most successful in each environment (Ruiz-Mirazo et al. 2014, de la Escosura et al. 2015).

Thus, the history of organelles and organs is mostly based on the evolution of proto-organs. An example is the origin of proto-ribosomes and ribosomes, organelles that gave rise to coded proteins and thus marked the end of the RNA world (as traditionally considered) (Fox et al. 2015). Once the genotype and phenotype were decoupled in RNA and proteins, respectively, the next major transition was the origin of DNA as an improved genetic material. These processes allowed for the advent of modern cells, in which the flow of genetic information was already established as DNA→RNA→proteins. Further evolution of them gave rise (among other branches) to the last universal common ancestor (LUCA) of all living beings ca. 3.8 billion years ago, a prokaryote or, perhaps, a community of them which then diversified into bacteria and archaea (Woese 2002).

As evolution proceeded across, certain organisms became organs in their turn. The paradigmatic example is the mitochondrion, which originally was a free-living bacterium (likely, an alphaproteobacterium) converted by endosymbiosis into an essential organelle of eukaryotic cells ca. 2.0 billion years ago, as studied in depth by Lynn Margulis (Sagan 1967, de Duve 2007). Later on, other organs essential for some eukaryotic lineages (plastids of photosynthetic algae and plants) originated by endosymbiosis of cyanobacteria (Long et al. 2024). Additionally, *Buchnera aphidicola*, an endosymbiotic bacterium of aphids, is a well-known example of how independent prokaryotic cells are currently becoming organelles of insect cells (Moya et al. 2008). Endosymbiosis-like processes might also occur in prokaryotic cells, explaining the long-term association of repetitive, extragenic, palindromic sequences (REPINs, noninfectious, nonautonomous entities that replicate within bacterial chromosomes; Bertels and Rainey 2023).

A similar case, in a much more advanced hierarchical level in biology, is that, in one of the eukaryotic branches of the Tree of Life in which animals were diversifying, the origins of the liver occurred 520 million years ago, giving rise to the first nonvertebrate chordates: amphioxi, also known as lancelets. In turn, in the branch that led to most mammalian animals, the placenta is an organ that originated probably ∼150 million years ago, likely due to the infection by a virus (perhaps a retrovirus), which led to the formation of syncytialized trophoblasts essential in the new organ (Chuong et al. 2013). Finally, the microbiota of humans (and other multicellular eukaryotes) can be considered a vital organ; however, the formation of such a complex organ is not the result of the expression of any information coded in the host genome, but of the reproduction of the individual (though interrelated) microbial species that compose it, once they were acquired from the progenitors and other external sources (Baquero and Nombela 2012, Dhanaraju and Rao 2022). In sum, it is worth considering that there is alternative, nongenetical, and interactive information in a “multidimensional Darwinism” (Baquero 2014).

These selected examples of the diverse relationships between organs and organisms along the Tree of Life certainly suggest that the chicken-and-egg paradox applies here. Therefore, is there a possibility for the existence of organs and organismal functions without organisms? Given the origin of the word organ, we could approach the question as if in the prebiotic (precellular) world, there were organ or organelle-like functions without organisms, devoid of any immediate biological function or use in cooperation with others. However, in the absence of cellular reproduction processes that guaranteed their evolutionary continuity, most of these prebiotic functions were ephemeral, contingency being compensated by their presumably high frequency of (re)emergence and (re)production.

## Organs without organisms

As mentioned earlier, primitive molecular assemblies “individuals” or organs might interact with others, thus improving the possibility of escape from annihilation. The more complex the ensembles, the higher the individuality level of the assemblies, so that the individual increases its hierarchy, internal interactions, and thus robustness. Here, robustness can be defined as the maintenance of a system despite fluctuations in the behavior of its component parts or its environment. However, “robust-yet-fragile” behaviors can be tolerated, which remain invisible to natural selection (Carlson and Doyle 2022).

The question now is whether the most primitive types of noncellular organs can perform any function besides their selfpreservation (robustness), thus influencing the external molecular environment. Such a function is exerted in the absence of any organismic integration, being apparently devoid of any Monodian or Pittendrighian teleonomy, that is, of any internal, inherent goal-seeking directness (Monod 1971, Corning et al. 2023). A particular molecule or macromolecular assembly could have found a function as, e.g. catalyzing a biochemical reaction. Without catalysts, molecular interactions among the reactants can occur if they are thermodynamically favored, though at an extremely low rate. Catalysts increase the rate of a reaction by lowering its activation energy, thus reducing the time needed for the conversion of reactants to products over a million-fold (e.g. from years to fractions of seconds). Given that we are celebrating in 2024 the centenary of the first work that scientifically addressed the chemical origin of life, by the Russian biochemist Alexander I. Oparin (Oparin 1924), it is worth remembering that the same author, in a later paper entitled “The Origins of Life and the Origins of Enzymes,” considered enzymes as “instruments” (i.e. organs, in the current proposal) and mentioned the Dixon and Webb statement: “We may surely say of the advent of enzymes… [it] was the most improbable and the most significant event in the history of the Universe” (Oparin 1965).

Proteins are the typical enzymes in current biology. However, the first catalysts were not proteins, to have been formed spontaneously from the available amino acids in the prebiotic world. In turn, it is assumed that the first catalysts operating in the transition from chemistry to biology were certain metals or mineral surfaces, followed by catalytic RNAs and RNA-peptide complexes (Ruiz-Mirazo et al. 2014, Frenkel-Pinter et al. 2020, Fine and Pearlman 2023). Inorganic catalysis occurred before the appearance of the first ribozymes and catalytic peptides, and indeed this is what allowed their origin by enabling the formation of covalent bonds between nucleotides or amino acids. A possible solution to this challenge is adsorbing the reactants on mineral surfaces or interlayers and then carrying out the condensations during a drying process (Kaddour et al. 2018) In a recent example of the use of such a strategy, Akouche et al. synthesized adenosine monophosphate from adenosine, ribose, and potassium dihydrogen phosphate on amorphous silica: it is assumed that primitive precellular ATP, the most important energy reservoir, could have had a similar origin (Chu et al. 2022).

The analysis of sequence-based evolutionary trees has suggested that one of the first protein enzymes, appearing at least 3.6 billion years ago, was adenylate kinase, which is present in every life form on Earth (Kerns et al. 2015). The birth of protein catalysts and the progressive increase of their performance (including the affinity for their substrates, the speed of the reactions catalyzed, their specificity, and their sensitivity to inhibitors or regulatory molecules) exponentially accelerated the evolution of macromolecular proto-organs and, ultimately, organ formation in primitive microorganisms. The chemical reservoir of energy, ATP, was probably critical in the construction of proto-organs, facilitating the confluence of biochemical and mechanical processes. A few examples of key microbial proto-organs with quite distinct functions are presented below: ion pumps, contractile filaments, ribosomes, and nucleic acids.

Proton-pumping ATPases are found in the three domains of life (Gogarten and Taiz 1992), and they are likely derived from simpler transmembrane pumps. Since their origin, they might have exerted a key effect in creating concentration-based interfaces as well as electrochemical gradients which led to the production of ATP, as proposed by the chemiosmotic hypothesis (Mitchell 1961). Pumps associated with multimolecular surfaces or vesicles can facilitate molecular interactions, eventually triggering the stabilization of macromolecular complexes (Mandal et al. 2024), emerging on multiple occasions during the course of evolution (Sliwinski 1998). Contractile motor filaments, as such derived from filamentous actin (F-actin) and myosin, provide a very intuitive image of an early “mechanical function” driven by ATP hydrolysis, eventually involved in macromolecular integration and vesicle replication (Sakamoto and Murrell 2024).

Concerning the origin of nucleic acids, the canonical hypothesis within the framework of the “RNA world” model, or its current, more plausible version known as the “RNA-peptide” world is that in the first step, RNA was formed (Biscans 2018, Xu et al. 2020) and copied itself. Condensation reactions on surfaces or liquid interphases have promoted chemically activated or cyclic ribonucleotides, giving rise to short RNA oligomers that later could give rise to longer and more functionally complex RNA molecules (e.g. by means of stepwise, ligation-based modular evolution (Briones et al. 2009, Manrubia and Briones 2007). The RNA-protein world came once the first ribosomes were fully functional, and the DNA–RNA-protein world evolved from it (Ruiz-Mirazo et al. 2014, Joyce and Szostak 2018). In the case of DNA, deoxyribonucleotides could be formed by means of the condensation of prebiotic canonical purine and pyrimidine nucleobases with acetaldehyde and a sugar-forming precursor, and they could then react with apiose (a branched sugar) to give rise to information-bearing polymers that could have preceded DNA (Teichert et al. 2019, Fialho et al. 2020). Another possibility is that DNA originated from previously formed RNA, when some ribozymes (or protein enzymes) with RNA polymerase activity mistakenly took deoxyribonucleotides available in the medium (instead of ribonucleotides) as monomers during the template-dependent replication of an RNA strand, thus giving rise to the first reverse transcriptases (Ruiz-Mirazo et al. 2014, Samanta and Joyce 2017) As it will be mentioned for proto-ribosomes, a large number of possible polymers (more or less) analogous to nucleic acids might have been formed by the combination of an immense variety of sugars, nucleobases, and linker molecules likely available in the abiotic environment (Fialho et al. 2020, Mikkola 2020, Devine and Jheeta 2020). However, the repertoire of nucleic acid analogs that could have populated the prebiotic world collapsed with the advent of proto-cellularity, and only RNA and DNA were preserved in cells. Among them, RNA was likely maintained as an intermediary molecule between DNA and proteins during the flow of genetic information thanks to its unsurpassed structural plasticity and functional versatility. In turn, the structural robustness of DNA (with two helical polymers wrapped around one another) made it a very stable archive of genetic information (Mills and Hagerman 2004), Mohammad-Rafiee and Golestanian 2004).

The evolutionary history of other proto-organs, such as the proto-ribosomes that preceded microbial ribosomes, is currently unknown. However, we can assume that these putative proto-ribosomes endowed with peptidyl transferase activity could selfassemble by the sequential interaction of a limited number of RNA molecules and (abiotically synthesized) peptides, resulting in the successive formation of biomolecular condensates that progressively optimized their efficiency and accuracy (Timsit et al. 2021, Bose et al. 2022, Farias-Rico and Mourra-Díaz 2022, Jaberi-Lashkari et al. 2023). This nascent architectural process might have occurred not only inside the cell, but it can also be promoted outside of the cellular environment (Levy et al. 2020), which suggests the possibility of a cell-independent existence of the first proto-ribosomal particles (Merl et al. 2010). In the absence of any specific and selectable biochemical function (beyond selfpreservation), myriad “robust” RNA-peptide (and, later on, RNA-protein) condensates could have been formed, giving rise to a growing repertoire of coacervates with different degrees of individuality (Agrawal et al. 2024). It is expected that only a very limited fraction of those macromolecular assemblies could have shown the right composition and functionality to be considered proto-ribosomes and, therefore, ancestors of the ribosomes. This “secondary selection” should be related to the emergence of a useful organismic function in cellular life: in this case, protein synthesis based on the RNA sequence, which was fundamental for genotype–phenotype decoupling.

Most probably, the molecular composition and structure of current pumps, contractile filaments, nucleic acids, and ribosomes in the current biosphere is simply the result of an extremely stringent series of bottleneck events produced along the evolution of proto-organs resulting from a huge number of macromolecular assemblies captured by primitive vesicles (Fang et al. 2024). From this incommensurable number of possible existing proto-organs and organs in the precellular environment, very few of them survived the selective pressures of natural selection and were maintained once cellular life originated. This does not imply that particular selected proto-organs are necessarily present in current organisms, as they could have been replaced (following a kind of proto-organs transfer) by the more fit ones in the organism.

## Proto-cellularity and organs’ evolutionary maturation

The random capture of various abiotic proto-organs at different relative concentrations by primordial vesicles was probably a critical step for the origin of life. The first selfassembled membranes were composed of amphiphilic compounds such as fatty acids and fatty alcohols, leading to the formation of micelles and closed vesicles (Deamer et al. 2002). The resulting compartmentalization of random ensembles of proto-organs in vesicles provided a heterogeneous combinatorial material for the selection of the fittest (i.e. robust or interactive) prebiotic assemblies (Cornejo et al. 2014). In these compartmentalized molecular landscapes of proto-cells, “molecules that stay together, evolve together,” according to Orgel's (2004) postulate. An important point is the heterogeneous stability of these vesicles, depending on the local conditions (Engberts and Kevelam 1996), such as local temperature, pH, determinants of vesicle size, and geometry (can be disk-shaped or cylindrical) so many proto-cells will never survive enough to be able to evolve. However, in particular environmental conditions, a few of these proto-cells might persist and be considered the uterus of life, thus initiating an evolutionary-developmental process based on the benefits of the interactions among the functions of the enclosed proto-organs. This process might have led to proto-organ maturation, changes in function (exaptation), coevolution, or cellular biogenesis ultimately giving rise to a particularly successful composition, a kind of “internal composome” (Kauffman 1993, Segré et al. 2000, Mulkidjanian et al. 2009, Caliari et al. 2021, Kahana et al. 2023).

The selfreplication of vesicles containing different compositions of proto-organs, following graded autocatalysis, and the complexification of some of them (as well as the internal proto-organ interactions) by fusion processes are conceivable in this prebiotic scenario. Although those vesicles containing proto-organs were still “infrabiological” lacking both genetic information and metabolism (Ruiz-Mirazo et al. 2014, Ruiz Mirazo et al. 2017), those processes should lead to significant increases in system information concerning dynamics (e.g. rates of synthesis and degradation of molecules or their aggregates), space (e.g. proximity, interactions, and shape complementarity), energy (e.g. charge distributions, both internal and across the membrane), and, in more complex systems, basic control mechanisms (e.g. regulation of the interactions) (Mayer et al. 2024, Chatterjee and Yadav 2022). It has been proposed that durability and selfreplication are contingent on the plasticity of metabolic processes (“metabolic plasticity”) (Seebacher and Beaman 2022). Once selfreplication and breakage–fusion cycles began in the population of vesicles containing proto-organs (in our model, the primitive organisms), a new, strong natural selection process could be initiated, which favored the fittest ones and led to the extinction of all other existing combinations of proto-organs. However, depending on the content of the newly formed vesicles, some proto-organs could also acquire a “new function” (exaptation, where a function is converted into another one) or spandrel functions (where a structure that apparently lacks functionality becomes a critical leftover to maintain an ensemble) by synergistic associations with other proto-organs, as has been observed in living organisms. For example, the precipitation of iron nanoparticles in association with organic material could have found a function as a mechanism able to mitigate the damaging effect of reactive oxygen species on primitive macromolecular structures (Lin et al. 2020).

## Emerging functions and the archeology of life

Although we have focused this hypothesis paper on the origin of the first organisms based on the meaningful assembly of prebiotic proto-organs, we should mention that current research in molecular evolution and genetics associated with the origin of life is also inspired by other evolving systems, as proposed in the emerging field of “RNA archaeology” (Ariza‐Mateos et al. 2024). Following experimental work on ancestral, functional RNA molecules and their progressive integration in coding RNA polymers (which would contribute to the construction of the first genomes operating in the RNA world), most of these primordial “tools” (including ribozymes) likely lost their original function and, thus, can be considered the “forgotten” or the “loser” elements in the evolutionary process (Ariza-Mateos et al. 2019, 2024).

In archeo-anthropology, ancient “tools” used by nonhuman primates were natural objects without any anthropogenic function, such as stones, shells, or fallen tree branches. These objects found a function (a “service function”) in the context of human evolutionary development, such as hammer stones, cutting shells, or sticks (Cummins-Sebree and Fragaszy 2005). Note that “tools” or “instruments” can again be considered a type of “extracorporeal proto-organ” providing new functions (capabilities) to the evolving humans. Thus, a “technical object” is the end product of an evolution, and it is something that cannot be considered a s amere utensil (Simondon 2011). Later on, the sequential assembly of these proto-organs (such as a stick with a stone at the end, functioning as a weapon) could have led to more complex “organismal” functions. Certainly, that recalls “metabolic collaboration” in living things (Dupré and O'Malley 2009, Dussault and Bouchard 2017).

Similarly, a critical advance in human evolution was the origin and diversification of language, a topic already treated by Darwin in his book “The Descent of Man” (Darwin 1871). Language function probably followed spontaneous (noncommunicative) signs and sounds produced by the individuals, which “found a social function” in early human communities and exponentially increased their semiotic significance when such signs and sounds were sequentially and deliberately associated with provide meaningful information (semantics), although in quite different languages (Kendon 2008). By analogy, sequentially built molecular assemblies opening the way to complex biomolecules, genetic polymers, and proto-organs could also be interpreted as primitive elements (signs) that allowed future communication strategies in the biological language(s) (Ariza‐Mateos et al. 2024). We are using these anthropological (societal drivers) metaphors to emphasize the role of succession in evolution, particularly that operating in “landscape contexts” that strongly impact successional pathways (Poorter et al. 2023). As in the former examples, proto-organs, organs, and organisms could have evolved asymmetrically in different times and environments of the Earth where the primordial composing elements were more or less abundant, and variable conditions for their interaction were present. There should be an “organismal geography” in the dawn of life (Buckley 2009).

## Transition probability: the odds of life

The probability that the first cells originated as a result of a successful combination of an unaccountable number of independent, prebiotic molecules or aggregates captured by primitive vesicles is impossible to determine; however, this probability should be extremely low. For instance, as previously noticed, the abiotic polymerization of ribonucleotides on mineral surfaces is not expected to have directly produced RNA oligomers long enough to perform complex biochemical functions, and further steps of molecular evolution are required (Manrubia 2022). Similarly, random polypeptide synthesis fed by the amino acids available in the prebiotic world had the potential to initially produce only an extremely small fraction of polypeptides that can fold spontaneously into catalytic domains (Kurland 2010).

Our proposal is based on the assumption that the number of macromolecular proto-organs that originated along a process of (still nonbiotic) natural selection and were captured by vesicles was necessarily extremely low. As previously stated, intravesicle maturation and complexification of organs from their macromolecular components remain compatible with this view. Therefore, “captured” RNA and polypeptide systems might have been the precursors of the cellular ribonucleoprotein (RNP) world that evolved subsequently, though this statement remains controversial (Kurland 2010)

**Figure 1.**
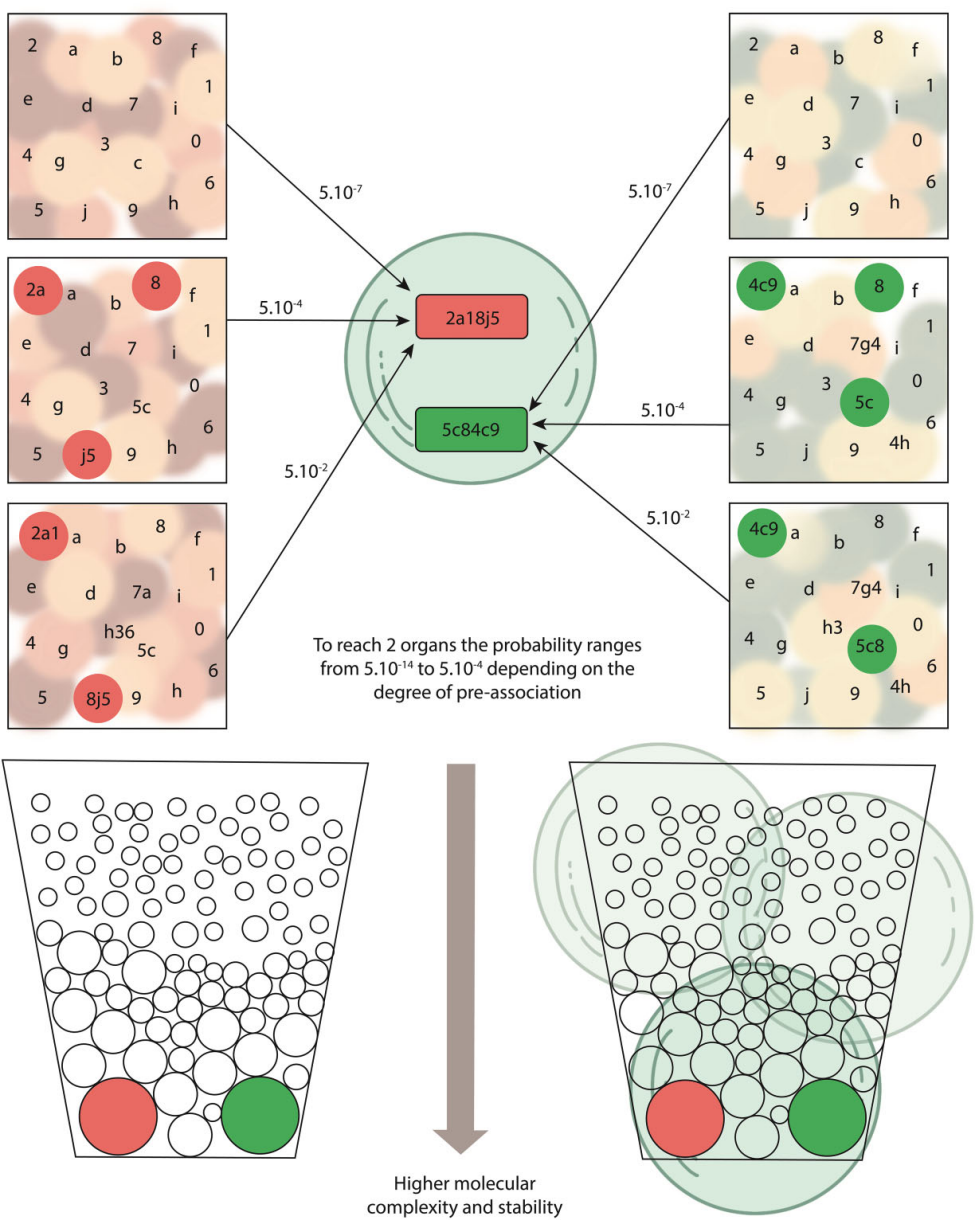
A highly simplified scheme showing how vesicles in light blue capture simple entities or more complex preformed ensembles of red and green small circles that should have emerged from molecules with lower levels of complexity. In the upper panel, in different prebiotic environments squares, some of these entities composed of one, two, or three members represented by numbers and letters will become part of selfconstructed, more complex, and six-membered shaped ensembles combining numbers and letters. Such ensembles might acquire some primitive functions, such as ensuring their permanence in time or modifying the environment, and are considered proto-organs red and green rectangles. The possibility that, by capturing only simpler entities, a proto-organ will develop inside a vesicle should be extremely low. In the center of the upper panel, a vesicle captures with the same probability various simple molecules or relatively complex proto-organs. The larger the number of captured components of proto-organs, the higher the probability for the emergence inside a vesicle of a six-member proto-organ. The possibility of having two or more different proto-organs in the same vesicle, and the resulting interactions between them, will contribute to the organ’s functional maturation and the vesicle will evolve as a proto-organism. In the lower panel, the larger associations of proto-organs tend to cluster in space and time, depending on their stability in particular environments, in such a way that they increase their possibility of being captured by the same vesicle forming big red or green circles, proto-organisms. The probabilities shown apply only to the number of elements in this schematic figure and are presented exclusively as an example: in the natural world, the differences in probability should be much higher in favor of the capture of proto-organs by vesicles, thus facilitating the origin of proto-organisms.

Even in this case, our hypothesis implies that most of the proto-organs were lost forever during the process, perhaps because the membrane compartments never captured them or, if they did, because their structural/functional requirements were incompatible with those of other proto-organs previously present in the vesicle. Only for probabilistic reasons should these elementary proto-organs and organs precede organisms (Fig. 1). This view reinforces the possibility that there may be alternative life forms to the only one we know of (derived from the first cells and LUCA) and thus encourages the search for other living entities (either artificial or extraterrestrial), both biochemically similar and different from the (extinct or extant) organisms we know. Therefore, our proposal could also be of interest in the fields of synthetic biology and astrobiology.

## Hypothesis should be tested

The hypothesis championed in this work, focused on the transition from prebiotic chemistry to the emergence of biochemical functions, proto-organs, organs, and organisms, appears intuitively and probabilistically sound; however, the question is whether it is experimentally testable. Hypotheses must be tested, not only championed, and ours could open new avenues for future research on the origin of life, complementary to the currently explored ones (Scharf et al. 2015, Preiner et al. 2020). However, it is out of the scope of this work to propose particular experiments to be carried out in the future. We limit ourselves to enumerating the main areas of research where our hypothesis could be tested. Approaches to this goal could come from prebiotic systems chemistry, including studies in dynamic combinatorial chemistry, selfassembly and selforganization, chemical selection, and autocatalytic systems (Ruiz-Mirazo et al. 2014). Also, clues to test this hypothesis could derive from technological advances in the fields of synthetic chemistry and nanotechnology, leading to the construction of new molecules and aggregate (Cooper et al. 2023), in chemo-informatics, including machine learning applied to combinatorial polymer chemistry, as well as in computational chemistry for predictive insights into chemical interactions (Keith et al. 2021). Also, quantum mechanics, based on the concept that physics constitutes the basics of biology, has been proposed as a field enlightening the origin and evolution of life (Torday 2018).

The final goal is the investigation of associative networks of interacting molecules and their aggregates in heterogeneous physicochemical environments and at various hierarchical levels to give rise to emergent functions in compartmented media (Altamura and Fiore 2022). A relevant hurdle to be crossed is the study of the compatibility between the physicochemical principles that control the natural selection operating in these material systems with the genotype–phenotype interdependence that drives Darwinian evolution (Danger et al. 2020, Manrubia 2022). The later steps in this bottom–up process would be the construction of synthetic (artificial) vesicles containing genetic and metabolic machineries, able to reproduce and evolve (thus, true living beings), though not necessarily mimicking the known forms of life (de Lorenzo and Danchin 2008, Blain and Szostak 2014). However, even the most promising scenarios for the *in vitro* development of vesicle populations would face tremendous bottlenecks in terms of preserving the complexity that, occasionally, might come out of their underlying chemistry (that could somehow selfproduce, grow, and reproduce), transferring various properties to the offspring (Ruiz-Mirazo et al. 2014, Segré et al. 2000). Also, pure evolutionary research may not provide all answers: development constraints, a subcategory of phyletic restrictions, may be critical in the directionality of the possible evolutionary pathways (Gould and Lewontin 1979). Many evolutionary pathways and functional trajectories are simply unpredictable, as they may follow a chaotic kinetics (Baquero et al. 2021).

In any case, we trust that future research lines could be useful to provide data in support of our hypothesis. The role of modern science is to overcome the challenge of complexity, as the evolution of ordered complexity is the epistemological backbone of general biology. It is really striking how similar the problems of life's origins are to the origin of any biological innovation. All the major transitions in evolution have occurred outside the most inclusive crown clade that possesses the targeted feature, meaning that its origin was much earlier than can be derived from empirical (e.g. genetic, physiologic, or paleontological) data. Novelty in biology is always the product of preexisting features that acquire a novel organization or function. Evolutionary processes and biological innovation come together, from the dawn of life to the current biosphere. In other words, life originated and diversified across successive steps of synthetic complexification, recalling the anthropogenic efforts in synthetic biology.

## Bioengineering echoes to an extent evolution's complexity-building process

The conceptual framework discussed above influences how contemporary synthetic biology aims to construct complex biological systems, including living cells, using the abstraction hierarchy and descriptive language typical of mechanical, electrical, and computational engineering (Garner 2021). The crucial point in both evolution and rational design is when a new function emerges from the combination of simpler, preceding components. As argued earlier, such a critical occurrence first happened in nature when specific molecular assemblies separated from the larger molecular pool to form what we have called a proto-organ, which was more stable and durable than other possible associations in its particular environment.

**Figure 2.**
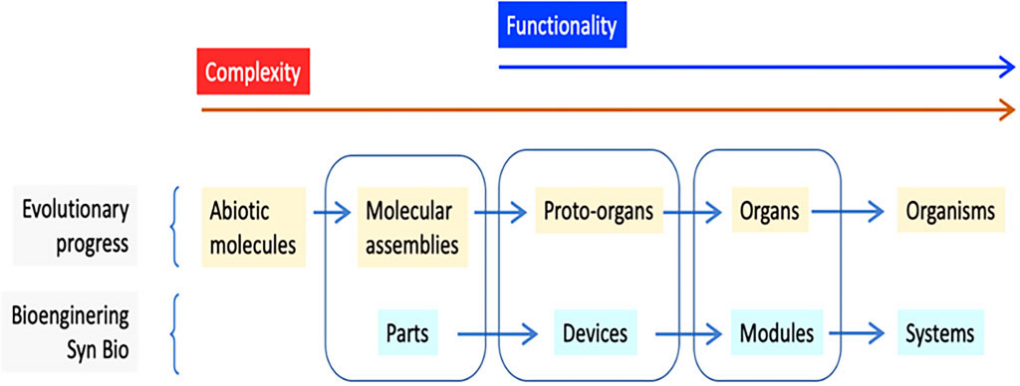
Equivalences between categories in naturally occurring Biological Evolution and the stepwise abstraction hierarchy of synthetic biology based on Andrianantoandro et al. (2006). Note the match among the three central classes but the divergence at the left and right sides. Genuine biological functionality starts in proto-organs/devices, although it is preceded by the accumulation or capture of molecular assemblies/parts of lower complexity. Note that in certain cases, organisms might become organs as in the case of mitochondria, and probably systems can act as modules of a higher level system.

It is important to note that the term organ applied to these assemblies denotes a certain level of organization (both physical and operational) and the performance of a specific task or function. In this context, a proto-organ is analogous to what is referred to as a “device” in synthetic biology terminology (see Fig. 2). The difference lies in the fact that the specific configuration of an evolutionary proto-organ is selected by the environment, whereas the construction of a device and the type/degree of separation from its context was chosen deliberately by a designer. Despite this distinction, both ways to deliver a particular action can result in similar or even identical outcomes, as demonstrated by the frequent instances of convergence between evolutionary solutions and rational design (de Lorenzo 2018).

As illustrated in Fig. 2, the next evolutionary step involves the transition from one or more molecular proto-organs to the formation of an organ. This occurs when the selected molecular assembly gains the ability not only to sustain itself but also to perform a specific function beyond mere persistence. For this to happen, the resulting organ must be integrated into a broader environment yet maintain its individuality, and fulfill a more complex function. In the context of synthetic biology, this is roughly equivalent to a “module,” which is a defined unit of connected devices that autonomously execute one or more operations within a biological framework.

However, evolution and bioengineering apparently diverge at the step that follows the formation of organs/modules. In the first case, the assembly of organs leads to the emergence of organisms, i.e. bona fide living entities capable of autonomous selfreproduction and propagation in time and space. In synthetic biology, however, the next and final phase in the process is the generation of a “system” (Andrianantoandro et al. 2006). This term encompasses a wide range of material scenarios that deliver complete and defined activities of various types, such as computation or catalysis. Generally, these engineered systems need to be implanted in an already existing biological host (a “chassis” in their own jargon) and thus do not yet achieve the status of living entities comparable to those resulting from naturally occurring evolution. In turn, in the biological world, when life was originated no chassis could be available as such. However, coming back to the field of synthetic biology, we can consider the existence of a “proto-chassis,” which could eventually evolve into an emergent chassis upon the acquisition of a new module, thus giving rise to an integrated organism-like system, already able to import and express new modules, and therefore to accelerating the evolutionary possibilities of the new constructions (networks).

These considerations highlight both the opportunities and challenges of adopting evolutionary strategies for the multiobjective optimization of complex artifacts produced by synthetic biology. While typical adaptive laboratory evolution (Mavrommati et al. 2022) or synthetically primed adaptation (Dvořák et al. 2024) strategies apply selection pressures to the semifinal constructs within their ultimate biological context, the ideas on complexity building discussed here suggest that such pressure should instead be applied at lower echelons of the construction process.

Our hypothesis is fully aligned with the view that modern life originated from a gradual process of chemical and biological evolution involving a series of transitional forms that connected prebiotic chemistry and LUCA (Ruiz-Mirazo et al. 2014, Fine and Pearlman 2023). This process implies the condensation of macromolecular entities and the progressive natural selection of the most performant of these condensates, either as autonomous functional units or as a result of an advantageous interaction with others (Caetano-Anollés et al. 2018). Within this framework, small isolated populations of ordered biomolecules constitute “nests” or “islands” that are in some sense “alive” (Dyson 1982), that is, have an ordered selfish or coordinated form–function. Disorder (death) is incompatible with function. In summary, in our view, biomolecules, molecular assemblies, functional molecular assemblies (proto-organs), organs, and organisms constitute the ascendant hierarchical succession of entities giving rise to the ultimate coordinated ensemble of functions that, in their most advanced term, at the end of its origin or “dawn” of what we designate as life.
